# Comparison between the modified long gonadotropin-releasing hormone agonist protocol and the non-downregulation protocol in POSEIDON groups: a propensity score matching retrospective cohort study

**DOI:** 10.3389/fendo.2023.1189357

**Published:** 2023-11-09

**Authors:** Chunyan Chen, Xinliu Zeng, Hanke Zhang, Qiongqiong Wei, Ying Gao, Lin Liu

**Affiliations:** Department of Obstetrics and Gynecology, Union Hospital, Tongji Medical College, Huazhong University of Science and Technology, Wuhan, China

**Keywords:** modified long gonadotropin-releasing hormone agonist protocol, nondown-regulation protocol, propensity score matching analysis, POSEIDON patients, ovarian hyporesponsiveness

## Abstract

**Background:**

*In vitro* fertilization (IVF) is the main technique to address the infertility issue in the patient-oriented strategy encompassing individualized oocyte number (POSEIDON) population. Adopting appropriate protocols for assisted reproduction technologies (ART) cycles in the POSEIDON group may attain more favorable pregnancy outcomes.

**Objectives:**

This study aimed to compare the effectiveness of modified long gonadotropin-releasing hormone agonist protocol and non-downregulation protocol in POSEIDON patients undergoing ART, and to identify the factors affecting the pregnancy outcomes in this group.

**Design:**

This study was designed as a propensity score-matched (PSM) retrospective analysis.

**Participants:**

The study cohort consisted of 910 patients diagnosed with ovarian hyporesponsiveness and treated by IVF from January 2020 to June 2022. They were followed up until the transfer of the last embryo of the IVF cycle and/or pregnancy at 12 weeks. The study was conducted at the Center of Reproductive Medicine, Tongji Medical College, Wuhan Union Hospital, Huazhong University of Science and Technology.

**Methods:**

The patients were divided into Group I and Group II. Group I was treated with modified long gonadotropin-releasing hormone agonist protocol while Group II was put on a non-downregulation protocol. Propensity score matching (PSM) was used to select patients for each group. The subjects were compared in terms of the baseline level, process of controlled ovarian hyperstimulation, and pregnancy outcomes. Binary logistic regression analysis was performed to assess the difference in the cumulative pregnancy rate between the two groups.

**Results:**

Of the 910 POSEIDON patients who underwent IVF, 213 received the modified long gonadotropin-releasing hormone agonist protocol and 697 were subjected to the non-downregulation protocol. From the original cohort, PSM matched 174 pairs of patients. No statistically significant difference was found in total gonadotropin (Gn) dose between the two PSM groups, but the average daily Gn dose was lower in Group I and the duration of Gn lasted longer. The number of retrieved oocytes, the number of metaphase II (MII) ooctyes retrieved, normal fertilization, and normal cleavage embryos was significantly higher in Group I than in Group II, but there existed no significant difference in the number of high-quality embryos between the two groups. The single-cycle CPR (cumulative pregnancy rate) was higher in Group I than in Group II (for Group I: before PSM, CPR = 52.6%; after PSM, CPR = 51.7%; for Group II: before PSM, CPR = 34.0%; after PSM, CPR = 34.5%), and the difference was statistically significant. A binary logistic regression analysis in the unmatched patients showed that the CPR of Group II was 0.486 times that of Group I (95% CI: 0.303 to 0.779).

**Conclusions:**

The modified long gonadotropin-releasing hormone agonist protocol can be used as an optimal protocol for IVF or ICSI (Intracytoplasmic sperm injection) in POSEIDON patients.

**Level of evidence:**

Level III

## 1 Introduction

Controlled ovarian hyperstimulation represents a very important part of assisted reproduction technologies (ART). Through controlled ovulation stimulation, infertile patients can generate sufficient high-quality oocytes, which are the premise of subsequent embryo culture and clinical pregnancy. Patients with ovarian hyporesponsiveness respond poorly to gonadotropin (Gn) during ovulation hyperstimulation, resulting in low oocytes retrieval and high cycle cancellation rate. Due to policy and social factors, many women experience a poor ovarian response(POR) when they want to have babies. The proportion of this population in assistant reproductive technology is increasing, which poses great challenges for clinicians. How to provide assisted pregnancy counseling to patients with low ovarian response and develop individualized assisted pregnancy strategies have become issues that have to be addressed urgently.

To better stratify the low ovarian response population and facilitate assisted pregnancy counseling, researchers proposed the low-response patient-oriented strategy encompassing individualized oocyte number (POSEIDON) criteria in 2016 (1). The POSEIDON criteria categorizes patients into four groups in terms of age, anti-Müllerian hormone (AMH), antral follicle count (AFC), and other indicators. The assisted pregnancy strategies and clinical outcomes vary with different groups of POSEIDON patients. However, no definitive consensus has been reached regarding how to formulate individualized ovarian hyperstimulation protocols for each POSEIDON group to attain maximal benefit (2–4).

Some protocols do not require pituitary downregulation and these include the antagonist protocol, the non-downregulation protocol, and the progestin-primed ovarian stimulation protocol. The advantages of these protocols lie in that they do not require pre-treatment, have a short cycle preparation time, and are economical and convenient. They are the most popular ovulation stimulation protocols with low-response patients. However, some therapeutic drugs involved in these protocols may disturb the endometrial receptivity (5–7). Most patients require whole embryo freezing and frozen-thawed embryo transplantation, but the low response patients tend to have fewer oocytes, a high risk of blastocyst culture, and a high cancellation rate of the transfer cycle. On the other hand, the mid-luteal-phase short-acting long gonadotropin-releasing hormone agonist (GnRHa) long protocol and the modified long gonadotropin-releasing hormone agonist protocol entail the use of GnRHa (gonadotropin-releasing hormone agonist) for pituitary downregulation. The treatment cycle lasts longer, the Gn dosage is high, and the number of oocytes retrieved is low. However, GnRHa can help improve endometrial receptivity (8, 9), and more patients can receive fresh embryo transfers. Until now, researchers have failed to agree on how to evaluate the efficacy of these two types of protocols. This study reviewed the *in vitro* fertilization cycles of patients with low ovarian response from January 2021 to June 2022 in our Center for Reproductive Medicine and comparatively examined the modified long gonadotropin-releasing hormone agonist protocol and the non-downregulation protocol with regards to their fertility-enhancing effect on the pregnancy result.

## 2 Materials and methods

### 2.1 Clinical data

Retrospective statistical analysis was performed on the data of patients who received IVF/ICSI (intracytoplasmic sperm injection) from January 2020 to June 2022 at the Center for Reproductive Medicine, Department of Obstetrics and Gynecology, Wuhan Union Hospital, Tongji Medical College, Huazhong University of Science and Technology, Wuhan, China. The eligible subjects for inclusion in this study were those who (1) met the relevant criteria of POSEIDON groups I–IV and (2) had received one of the two clinical protocols (i.e., modified long gonadotropin-releasing hormone agonist protocol and non-downregulation protocol) in our center, and had been followed up until the last embryo of the oocyte retrieval cycle was transferred or the pregnancy was over 12 weeks. Exclusion criteria for this study included (1): patients who had concomitant uterine malformation, refractory intrauterine effusion, or intrauterine adhesion (2); patients who were undergoing preimplantation genetic testing (PGT); and (3) those who had other medical and surgical conditions, such as hypertension, diabetes, and tumors. In this study, 910 patients who satisfied the aforementioned inclusion criteria included 213 patients on the modified long gonadotropin-releasing hormone agonist protocol (Group I) and 697 patients receiving the non-downregulation protocol (Group II).

#### 2.1.1 POSEIDON criteria

The POSEIDON criteria categorizes people with poor ovarian response into four groups.

POSEIDON group 1: Patients < 35 years with normal ovarian reserve parameters (AFC > 5, AMH > 1.2 ng/mL); however, they have an unexpected poor ovarian response.

POSEIDON group 2: Patients > 35 years with normal ovarian reserve parameters (AFC > 5, AMH > 1.2 ng/mL); they have an unexpected poor ovarian response.

POSEIDON group 3: Patients < 35 years with poor ovarian reserve parameters (AFC < 5, AMH < 1.2 ng/ml).

POSEIDON group 4: Patients > 35 years with poor ovarian reserve parameters (AFC < 5, AMH < 1.2 ng/mL).

### 2.2 Ovarian hyperstimulation protocol

This study was designed to compare the efficacy of two protocols, the modified long gonadotropin-releasing hormone agonist protocol and the non-downregulation protocol, in patients who met the POSEIDON criteria. Therefore, the ovarian hyperstimulation regimens in this study included the two above-mentioned protocols. The ovulation protocol employed the standard operating procedure (SOP) of the ovulation induction protocol of our center, and the procedure, detailed as follows:

#### 2.2.1 Modified long gonadotropin-releasing hormone agonist protocol

Patients took 3.75 mg of gonadotropin-releasing hormone agonist (GnRHa) during day 2 to day 5 of the menstrual period and returned to the clinic for further consultation on day 28 after the downregulation. The starting time was determined according to the follicle size and the blood hormone levels. When the follicles reached the right size for ovulation, the ovulation was triggered with recombinant human chorionic gonadotropin/human chorionic gonadotropin (rHCG/HCG) injection and the oocytes were retrieved after 36-40 hours.

#### 2.2.2 Non-downregulation protocol

The patients came to the hospital for ultrasound monitoring and hormone testing on day 2/day 3 of menstruation and the initiating dose of Gn was determined by the patient’s age, body weight, AMH, and other factors. At the same time, the patient took oral clomiphene citrate at 100 mg per day. When the follicles reached the optimal size, the patient was administered GnRHa 0.2 mg/rHCG 250-500 μg/HCG 4,000-10,000 IU, alone or in combination with the trigger injection, and the oocytes were retrieved within 36-40 hours. Fresh embryo transfer was not conducted with this protocol, and all embryos were cultured into blastocysts and then frozen.

### 2.3 Transfer strategy

#### 2.3.1 Fresh embryo transfer

Patients on a modified long gonadotropin-releasing hormone agonist protocol received luteal phase support (dydrogesterone tablets at 10 mg *po bid* and progesterone at 60 mg *im qd*) after oocyte retrieval. On the second day after egg retrieval, patients received type B ultrasound to measure endometrium thickness. If the endometrium was ≥ 7 mm, no uterine cavity effusion was found, and the diameter of both ovaries measured less than 70 mm, embryo transfer was performed on the third day post-oocyte retrieval. One or two embryos were transferred, depending on the embryo grade, and the remaining embryos were cultured to blastocysts and then cryopreserved.

#### 2.3.2 Frozen embryo transfer

The patients who did not become pregnant after the whole cycle of embryo freezing or fresh embryo transfer were subjected to the hormone replacement therapy (HRT) protocol and underwent endometrial preparation on day 2 of menstruation. For HRT protocol, oral estradiol valerate tablets were given at 4-6 mg/d, starting on day 2 of menstruation. After 10 to 15 days, when the endometrial thickness was more than 7 mm, blood samples were taken for estradiol and progesterone determination. When serum estradiol (E2) ≥ 200 pg/ml and serum progesterone (P) <1.5 ng/ml, progesterone was given to transform the endometrium. On the sixth day of endometrial transformation, the uterus was rechecked sonographically. When the endometrium reached a thickness greater than 7 mm, the embryos were thawed and transferred.

### 2.4 Outcome measures

This study compared the outcomes of the modified long gonadotropin-releasing hormone agonist protocol and the non-downregulation protocol in POSEIDON group patients by utilizing retrospective analysis plus propensity score matching (PSM). We matched the baseline conditions of the two groups of patients using PSM and then compared the ovulation and pregnancy outcomes of the two groups. The main outcome indicators covered by this study included:

#### 2.4.1 Major outcome measures

The major outcome was the cumulative pregnancy rate of a single cycle upon use of the two different ovarian hyperstimulation protocols in the POSEIDON group patients. Single-cycle cumulative pregnancy rate = the number of pregnancy cycles after fresh or thawed transplantation of the same ovulation cycle/total number of ovarian hyperstimulation cycles × 100%.

#### 2.4.2 Secondary outcome measures

The secondary measures included total amount of Gn, Gn days, the number of oocytes retrieved, the number of MII ooctyes retrieved, the number of high-quality embryos, and the number of frozen embryos.

### 2.5 Statistical analysis

The SPSS 25.0 software package was employed for statistical analysis. The basic indicators, such as the age of the male and female patients, number of stimulation cycles, fertilization methods, infertility factors, years of infertility, body mass index (BMI), AFC, and AMH, were used as covariates for matching. The tolerance was set at 0.05. A new dataset was created for the matched cases. To understand whether there existed statistically significant differences among the above basic indicators, a multifactorial binary logistic regression was performed on the indicators with significant differences, to understand whether the ovulation stimulation protocol impacted the pregnancy outcome in patients with ovarian hyporesponsiveness. 

The normality test was conducted on the measurement data, and the data that conformed to the normal distribution were expressed as mean and standard deviation (SD), and the independent sample t-test was used for statistical analysis. The data not following the normal distribution pattern were presented as median (the 25th percentile/the 75th percentile), and the comparison between groups was made using the Mann–Whitney U test. The enumeration data were reported as percentages (%), and the ratios of constituents between groups were comparatively analyzed by utilizing the Chi-square test. A *P* less than 0.05 indicated that the difference was statistically significant.

## 3 Results

### 3.1 Baseline data before and after matching in the two groups

The main objective of this study was to compare the pregnancy outcomes of two different ovulation stimulation protocols, i.e., the modified long gonadotropin-releasing hormone agonist protocol and the non-downregulation protocol in patients with low ovarian response against the POSEIDON criteria. The two groups were compared in terms of basic indicators including: the age of both the male and female patients, ovulation cycle(s), infertility years, BMI, AMH, AFC, POSEIDON grouping, infertility type, infertility causes, and fertilization technique used. The results are shown in **Table 1**. There were statistical differences in multiple baseline features between the two groups, suggesting that there existed a selection bias when clinicians chose protocols for patients with low prognosis.

**Table 1:** Comparison of baseline data before and after the matching of two protocols.

	Original data			P-value	Data after matching			P-value
	Group I (n=213)	Group II (n=697)	*T or χ*2		Group I(n=174)	Group II (n=174)	*T or χ*2	
**Female age**	33.11 ± 4.50	34.62 ± 5.18	-3.83	0.000^a^	33.762 ± 4.55	33.132 ± 5.35	1.20	0.232
**Male age**	34.092 ± 5.26	35.872 ± 6.20	-4.15	0.000^a^	34.612 ± 5.42	33.662 ± 5.59	1.62	0.107
**Stimulation cycle(s)**	1 [1/2]	1 [1/2]	2.20	0.028^a^	1 [1/2]	1 [1/2]	0.54	0.587
**Infertility years**	3 [1/4]	3 [1.5/4]	0.22	0.825	3 [1/4]	2 [1/4]	0.78	0.438
**BMI**	23.082 ± 3.61	23.162 ± 3.55	-0.28	0.778	23.032 ± 3.71	23.702 ± 4.06	-1.61	0.109
**AMH**	2.10 [1.12/3.56]	1.06 [0.61/1.58]	10.40	0.000^a^	1.69 [1.0/2.68]	0.92 [0.23/2.27]	5.24	0.000^a^
**AFC**	10 [8/13]	8 [6/10]	7.11	0.000^a^	9 [7/11]	9 [6/11]	2.71	0.007^a^
**POSEIDON grouping**			72.73	0.000^a^			1.15	0.765
Group 1	112	171			76	68		
Group 2	43	123			40	42		
Group 3	23	191			23	22		
Group 4	35	212			35	42		
**Infertility type**			5.11	0.024^a^			2.27	0.132
Primary infertility	107	289			86	101		
Secondary infertility	106	408			88	73		
**Causes of infertility**			10.37	0.016^a^			7.12	0.068
Female factor	170	596			141	158		
Male factor	14	28			12	5		
Couple factors	6	33			6	3		
Unknown reasons	23	40			15	8		
**Fertilization techniques**			23.84	0.000^a^			14.91	0.000^a^
IVF	137	338			106	130		
ICSI	68	318			61	44		
RICSI	8	10			7	0		

a. P<0.05.

BMI, body mass index; AMH, anti-Müllerian hormone; AFC, antral follicle count; IVF, in vitro fertilization; ICSI, intracytoplasmic sperm injection; RICSI, rescue intracytoplasmic sperm injection.

To compare the effect of two different ovulation stimulation protocols on the clinical outcomes, we used the propensity scoring to screen the data of two groups. With the aforementioned basic indicators as covariates and the tolerance set at 0.05, the subjects were matched at 1:1 and the unmatched cases were excluded. As a result, a total of 174 patients remained in each group. Post-matching comparison of the basic data between the two matched groups revealed that there were no statistically significant differences between the two groups in age, number of stimulation cycles, POSEIDON group, type of infertility, and the causes of infertility. Nonetheless, upon propensity score matching, there still existed a statistically significant differences between the two groups in terms of AMH, AFC, and fertilization techniques.

### 3.2 Comparison of ovulation stimulation protocols and clinical outcomes between the two groups before and after matching

The two ovulation stimulation protocols and their pregnancy outcomes before and after matching were comparatively analyzed, and the results are given in **Table 2**. The post-matching data showed that the total amount of Gn used by patients on the non-downregulation protocol was higher, the number of Gn days was lower, and the average daily dosage of Gn and Gn initiation doses were significantly increased. Compared with the modified long gonadotropin-releasing hormone agonist protocol, the number of oocytes obtained, the number of mature oocytes, normal fertilization, and normal cleavage were significantly lower in the non-downregulation protocol. However, the high-quality embryo rate of the non-downregulation protocol was comparable to that of the modified long gonadotropin-releasing hormone agonist protocol, and the number of frozen blastocysts was higher than that of the modified long gonadotropin-releasing hormone agonist protocol. These results might be ascribed to the fact that over 80% of patients on the modified long gonadotropin-releasing hormone agonist protocol underwent fresh embryo transfer. With the non-downregulation protocol, the whole embryo culture and blastocyst freezing were used after oocyte retrieval, so the number of frozen blastocysts was more than that with the modified long gonadotropin-releasing hormone agonist protocol. Patients with ovarian hyporesponsiveness had fewer eggs and substantially fewer embryos and it was very likely that no blastocyst developed in the process of blastocyst culture. This resulted in a 33.7% cancellation rate, which was significantly higher than the 11.7% cancellation rate observed with the modified long gonadotropin-releasing hormone agonist protocol.

**Table 2:** Comparison of ovulation process and clinical outcome before and after the matching of two protocols.

	Original/Pre-matching data			P-value	Data after matching			P-value
	Group I(n=213)	Group II(n=697)	*T or χ*2		Group I (n=174)	Group II (n=174)	*T or χ*2	
**Total Gn (IU)**	27,892 ± 1,014	31,652 ± 896	-3.66	0.000^a^	29,922 ± 1,055	31,932 ± 1,026	-1.48	0.139
**Gn days**	11 [9/12]	10 [8/11]	5.97	0.000^a^	11 [10/12]	10 [8/11]	5.25	0.000^a^
**Average daily Gn (IU)**	242 [187/279]	300 [292/355]	-14.93	0.000^a^	254 [206/286]	300 [291/347]	-9.88	0.000^a^
**Gn activation doses (IU)**	200 [150/225]	300 [300/375]	-21.31	0.000^a^	200 [150/225]	300 [300/375]	-14.29	0.000^a^
**Trigger day LH (IU/L)**	0.78 [0.57/1.35]	6.93 [4.69/10.27]	-18.52	0.000^a^	0.87 [0.54/1.30]	7.83 [5.47/11.69]	-7.221	<0.001^a^
**Trigger day E2 (pg/ml)**	881 [433/1219]	1,682 [1,057/2,397]	-11.11	0.000^a^	872 [383/1,192]	1,821 [855/2,505]	-13.53	0.000^a^
**Trigger day P (ng/ml)**	0.57 [0.40/0.85]	0.77 [0.50/1.15]	-4.99	<0.001^a^	0.55 [0.39/0.85]	0.86 [0.50/1.20]	-4.25	<0.001^a^
**Add GnRH-A ratio**		54/697 (7.75%)				9/174 (5.17%)		
**GnRH-A dose**		0.25 [0.25/0.50]				0.25 [0.25/1.0]		
**Premature ovulation ratio**		7/697 (1.00%)				2/174 (1.15%)		
**Retrieved oocytes**	6 [3/8]	4 [2/6]	5.42	0.000^a^	5 [3/8]	4 [2/7]	3.02	0.003^a^
**Mature oocytes**	5 [2/6]	3 [2/6]	3.96	0.000^a^	4 [2/6]	3 [1/6]	2.05	0.041^a^
**Normal fertilization**	3 [2/5]	2 [1/4]	3.80	0.000^a^	3 [1/6]	2 [1/4]	2.05	0.041^a^
**Normal cleavage**	3 [1/4]	2 [1/4]	3.83	0.000^a^	3 [1/4]	2 [1/4]	2.11	0.035^a^
**High- quality** **embryo rate of D3**	1 [0/2]	1 [0/1]	1.69	0.000^a^	1 [0/2]	1 [0/1]	0.90	0.368
**Frozen blastocyst**	0 [1/2]	1 [0/2]	-4.73	0.000^a^	0 [0/1]	1 [0/2]	-4.04	0.000^a^
**Cumulative pregnancy rate**	112/213 (52.6%)	237/697 (34.0%)	23.82	0.000^a^	90/174 (51.7%)	60/174 (34.5%)	10.55	0.001^a^
**Transplant cancellation Rate**	25/213 (11.7%)	235/697 (33.7%)	38.62	0.000^a^	23/173 (13.2%)	61/174 (35.1%)	22.66	0.000^a^
**Fresh embryo transfer ratio**	185/213 (86.9%)				148/174 (85.1%)			
**Average transfer embryos**	265/185(1.42)				212/148(1.43)			
Single embryo	107/185 (57.8%)				84/148 (56.8%)			
Double embryos	78/185 (42.2%)				64/148 (43.3%)			
	93/185 (50.3%)				76/148 (51.4%)			

a. P<0.05.

Post-matching data exhibited that there was no significant difference in the total amount of Gn used between the two protocols. The duration of Gn in the modified long gonadotropin-releasing hormone agonist protocol lasted longer, and the daily average Gn dosage and Gn initiation dosage were significantly lower than those of the non-downregulation protocol. After matching of the basic data, the number of oocytes retrieved and mature oocytes, the normal fertilization and normal cleavage with the modified long gonadotropin-releasing hormone agonist protocol were still significantly higher than those with the non-downregulation protocol, but there was no significant difference between the two groups in the number of high-quality embryos on day 3. After matching, the results still showed that the cumulative pregnancy rate of the modified long gonadotropin-releasing hormone agonist protocol was significantly higher than that of its non-downregulation counterpart. In the matched data, the transfer cancellation rate of the non-downregulation protocol, due to lack of embryo freezing, was also significantly higher than that of the modified long gonadotropin-releasing hormone agonist protocol.

### 3.3 Determination of the independent factors influencing the cumulative pregnancy rate

The baseline data of the two groups of POSEIDON group patients on different clinical protocols still exhibited significant differences after PSM matching. Statistical tests still showed significant differences between the two matched groups in AMH, AFC, and fertilization technique. We subjected the three factors plus the group factors to the logistic regression to see whether the clinical ovulation stimulation protocol was an independent factor influencing the cumulative pregnancy rate of POSEIDON group patients. As shown in **Table 3**, the ovulation stimulation protocol group was an independent factor influencing the cumulative pregnancy rate in POSEIDON group patients. The cumulative pregnancy rate of the non-downregulation protocol was 0.486 times that of the modified long gonadotropin-releasing hormone agonist protocol, with a 95% confidence interval of 0.303-0.779, and the difference was statistically significant. In addition to the clinical ovulation stimulation protocol, the number of basal antral follicles and the group of fertilization methods were also independent influencing factors. As the number of basal antral follicles increased, the cumulative pregnancy rate also increased, with the OR value being 1.128 and its 95% confidence interval 1.046-1.218, and the difference was statistically significant. With respect to the group of different fertilization methods, the cumulative pregnancy rate of ICSI (intracytoplasmic sperm injection) was lower than that of IVF, while the difference between RICSI (rescue ICSI) and IVF was not statistically significant. In this study, the proportion of ICSI and RICSI was relatively small. These results did not rule out the possibility of bias. In future, larger-sized studies are warranted for further verification.

**Table 3:** Regression analysis of factors influencing cumulative pregnancy rate in patient-oriented strategy encompassing individualized oocyte number (POSEIDON) group patients.

	B values	SD	P-value	OR	OR (95% CI)	
					5%	95%
**Protocol grouping**	-0.722	0.241	0.003^a^	0.486	0.303	0.779
**AMH**	0.164	2.697	0.101	1.178	0.969	1.432
**AFC**	0.121	0.039	0.002^a^	1.128	1.046	1.218
**Fertilization methods**			0.004^a^			
ICSI/IVF	-0.835	0.273	0.002^a^	0.434	0.254	0.741
RICSI/IVF	-1.521	0.889	0.087	0.218	0.038	1.248
**Constant**	-1.036	0.388	0.008^a^	0.355		

a. P<0.05.

AMH, anti-Müllerian hormone; AFC, antral follicle count; IVF, in vitro fertilization; ICSI, intracytoplasmic sperm injection; RICSI, rescue intracytoplasmic sperm injection.

## 4 Discussion

Controlled ovarian stimulation (COS) is a pivotal part of ART. Controlled ovarian hyperstimulation allows infertile women to yield a sufficient number of oocytes, which is important for attaining high pregnancy and live birth rates. Ovarian hyporesponsiveness is principally characterized by a poor response to Gn. With low responders, the dose of Gn used in the ovulatory cycle is high but the quality and quantity of oocytes are poor, resulting in low pregnancy rates and high cycle cancellation rates. In fact, POR poses a major challenge for ART. For the POR patients, it is particularly important to work out an ovulation stimulation protocol to each individual.

In 1984, Porter et al. (10), for the first time, successfully used GnRHa in combination with gonadotropin for ovulation induction. Since then, pituitary downregulation was extensively employed as ovarian hyperstimulation treatment in in vitro fertilization and embryotransfer (IVF-ET). GnRHa binds stably to the pituitary gonadotropin-releasing hormone receptor. After a short flare-up period, pituitary is functionally suppressed, follicle-stimulating hormone (FSH) and luteinizing hormone (LH) levels are reduced, and eventually pituitary function is effectively inhibited. Functional downregulation of the pituitary synchronizes the recruitment of follicles. Upon the downregulation, exogenous Gn dosing can achieve the synchronous and uniform the growth of follicles, thereby improving the outcome of ART. However, patients with low ovarian function already respond poorly to Gn medication. After downregulation and inhibition of endogenous Gn secretion, even with additional higher doses of exogenous Gn, follicular dysplasia or even aplasia may still occur. Therefore, our early practice was to treat most POR patients with a non-downregulation protocol. In this protocol, patients were given sufficient doses of Gn to promote ovulation during the menstrual period, with oral clomiphene citrate (CC) serving as adjuvant therapy. CC is a selective estrogen receptor modulator chemical. In general, it exerts predominantly an estrogenic antagonist or anti-estrogenic effect. As an anti-estrogenic agent, CC can act directly on hypothalamic gonadotropin-releasing hormone (GnRH) neurons and indirectly promote the release of GnRH by inhibiting the negative feedback of endogenous estrogens in hypothalamus. GnRH secreted enters the pituitary portal system, stimulating the secretion of pituitary FSH and LH, which stimulate ovarian activity, and promote the growth, development, and maturation of follicles and ovulation (11). A prospective, randomized, controlled trial by Al-Inany Hesham et al. demonstrated that the addition of CC to hMG could effectively reduce premature LH surges without compromising the pregnancy rate (12). However, this protocol may be associated with early onset of LH peak and early ovulation due to lack of downregulation. In order to prevent early ovulation, a GnRH antagonist is occasionally used if serum LH exceeds 10 mIU/ml during superovulation monitoring in the non-downregulated protocol. With the non-downregulation protocol, CC plus high-dose Gn entails no pretreatment, does not affect follicular development, and involves only a short stimulation time. Its use is more economical and efficient in patients with POR. However, due to the anti-estrogen effect of CC, which affects the growth and development of the endometrium, the endometrium of most patients on the ovulation stimulation cycle cannot satisfy the requirements for transplantation (13). Therefore, in using the non-downregulation protocol, we employ the “freeze all” strategy. All the embryos obtained by this protocol are cultured to blastocysts and frozen. In general, the ovarian response of POR patients is poor and fewer oocytes are retrieved. In particular, a significant number of patients are unable to have blastocysts available for transfer under the strategy. For the past two years, our center has been trying to use the modified long gonadotropin-releasing hormone agonist protocol for ovarian hyperstimulation in poor responders. The long-acting GnRHa in the modified long gonadotropin-releasing hormone agonist protocol can inhibit immune and inflammatory factors, upregulate the expression of the endometrial cell adhesion molecule integrin, enhance endometrial pinocytosis in the implantation window, and thereby improve endometrial receptivity (5, 6, 8, 9, 14). Patients with ovarian hyporesponsiveness have poor responses to Gn, the number of oocytes retrieved is low, and the number of embryos available is few. The long-acting GnRHa used in the modified long gonadotropin-releasing hormone agonist protocol can improve endometrial receptivity and the POR patients with this protocol potentially have more opportunity for fresh embryo transfer. This study showed that the modified long gonadotropin-releasing hormone agonist protocol could accomplish a fresh embryo transfer rate of more than 85%.

Due to the presence of selection bias, it is meaningless to directly compare the two protocols in terms of pregnancy outcomes. Therefore, our study performed a propensity score matching on the retrospective data and comparatively analyzed the two protocols in terms of outcomes. We found that although there were more frozen blastocysts with the non-downregulation protocol, its cumulative single cycle pregnancy rate was still significantly lower than that with the modified long gonadotropin-releasing hormone agonist protocol. This study suggests that the modified long gonadotropin-releasing hormone agonist protocol can be used as an option for individualized ovarian hyperstimulation in POSEIDON group patients. Nevertheless, although the single-cycle cumulative pregnancy rate of the modified long gonadotropin-releasing hormone agonist protocol is not an optimal alternative, it is still worth future investigation and exploration as a pregnancy strategy for low responders.

Other studies also examined the use of the modified long gonadotropin-releasing hormone agonist protocol in patients with low response. A retrospective study by Huang MC et al. (3) also suggested that the modified long gonadotropin-releasing hormone agonist protocol might have more advantages over the GnRH-antagonist protocol when used in young POR populations. The modified long gonadotropin-releasing hormone agonist protocol in a young POR population yielded a lower transplant cancellation rate and attained a higher implantation rate, which might be attributed to the improved embryo quality and endometrial receptivity. Another study by Guo Y et al. (2) suggested that low-response patients with normal AFC and low AMH levels might benefit from a modified long gonadotropin-releasing hormone agonist protocol. However, women with normal AMH but low AFC appeared to have more favorable clinical outcomes with the mid-luteal-phase short-acting GnRH-agonist long protocol. Li w et al. (15) compared the pregnancy promoting outcomes of 451 IVF/ICSI patients in the POSEIDON 3 group between June 2017 and June 2020 under three different ovarian stimulation protocols, and the results suggested that the cumulative pregnancy rate and cumulative live birth rate were significantly higher in patients using the modified ultra-long protocol than those using the antagonist protocol and the mild stimulation protocol (50.88% vs 32.02% and 31.88%, respectively, for cumulative pregnancy rate, and 48.25% vs 26.97% and 28.99%, respectively, for cumulative live birth rate). Women in the POSEIDON 3 group who underwent IVF-ET with the modified ultra-long protocol had higher stimulation duration and total Gn dose and thicker endometrial thickness. The findings suggest that the modified ultra-long protocol increases the cumulative pregnancy and cumulative live birth rates in women with a poor ovarian response in the POSEIDON 3 group. Currently, the selection of an individualized ovulation induction protocol for patients with a low ovarian response remains controversial. Bias can make the study results less consistent. The aforementioned studies attempted to reduce the impact of bias by stratifying them in terms of age or by subgrouping. In contrast, our study aimed to minimize the effect of bias between the two groups of patients by using PSM. This ensured the reliability of our results.

While our study provides data for the further exploration of the individualized ovulation induction protocol for treating patients with low response, it is subject to some limitations. Firstly, the sample size of the modified long gonadotropin-releasing hormone agonist protocol was small, making it difficult to make comparison between groups in terms of the POSEIDON groups. The exclusion of some cases for matching might lead to selection bias. Secondly, this study was a retrospective study. Despite the use of statistical methods to minimize bias, it was impossible to completely prevent bias. In future, larger-sized studies or prospective research are needed to further verify or modify the above conclusions.

## 5 Conclusion

In summary, the selection of ovarian stimulation strategies for patients with poor ovarian response has been a challenge. The modified long gonadotropin-releasing hormone agonist protocol improves the endometrial receptivity, increases the fresh embryo transfer rate, and reduces the cycle cancellation rate, giving patients more opportunities to receive an embryo transfer. The protocol accomplishes better clinical outcomes compared to its non-downregulation counterpart. Therefore, the modified long gonadotropin-releasing hormone agonist protocol can be an alternative individualized ovulation induction protocol for patients with ovarian hyporesponsiveness.

## Data availability statement

The raw data supporting the conclusions of this article will be made available by the authors, without undue reservation.

## Author contributions

LL and YG contributed to the paper design and writing, CC and XZ contributed to the data processing and paper writing, All authors contributed to data collection. All authors contributed to the article and approved the submitted version.
