# Supplementary material for: Comparison between the modified long gonadotropin-releasing hormone agonist protocol and the non-downregulation protocol in POSEIDON groups: a propensity score matching retrospective cohort study
Source: Front Endocrinol (Lausanne). 2023 Nov 9;14:1189357. doi: 10.3389/fendo.2023.1189357 (PMC10666785; doi:10.3389/fendo.2023.1189357)
Supplement: Supplementary file 1 [file Table_1.docx]

Supplementary table S1

Table S1

Multivariate logistic regression analysis of factors influencing cumulative pregnancy rate in Poseidon group patients

|  | B values | SD | P-value | OR | OR (95% CI) | |
| --- | --- | --- | --- | --- | --- | --- |
|  |  |  |  |  | 5% | 95% |
| **Protocol grouping** | -0.810 | 0.239 | 0.001^a^ | 0.445 | 0.278 | 0.711 |
| **Female age** | -0.152 | 0.044 | 0.001^a^ | 0.859 | 0.788 | 0.937 |
| **Male age** | 0.019 | 0.038 | 0.620 | 1.019 | 0.945 | 1.099 |
| **Infertile factors** |  |  | 0.297 |  |  |  |
| Male factor/female factor | 0.808 | 0.560 | 0.149 | 2.243 | 0.748 | 6.728 |
| Couple factors/female factor | 0.283 | 0.749 | 0.706 | 1.326 | 0.305 | 5.762 |
| Unknown reasons/female factor | 0.639 | 0.478 | 0.181 | 1.894 | 0.743 | 4.830 |
| **BMI** | -0.022 | 0.031 | 0.468 | 0.978 | 0.920 | 1.039 |
| **Infertility years** | -0.007 | 0.044 | 0.877 | 0.993 | 0.912 | 1.082 |
| **Constant** | 4.957 | 1.051 | 0.001 | 142.232 |  |  |

a. P<0.05

BMI: Body mass index.
